# Cognitive Frailty Among Elderly Chinese Patients With Cerebral Small Vessel Disease: A Structural MRI Study

**DOI:** 10.3389/fmed.2020.00397

**Published:** 2020-09-04

**Authors:** Hóngyi Zhào, Wei Wei, Yu Liu, Jiajia Gao, Yonghua Huang

**Affiliations:** ^1^Department of Neurology, Chinese PLA General Hospital, Beijing, China; ^2^Department of Neurology, Number 984 Hospital of the PLA, Beijing, China

**Keywords:** cognitive frailty, fried frailty phenotype, neuroimaging, magnetic resonance imaging, cerebral small vessel disease

## Abstract

Cognitive frailty (CF) is gaining an increasing amount of attention in geriatric research. CF refers to the co-occurrence of physical frailty and cognitive impairment in people without dementia. Neuroimaging of elderly people has revealed the presence of numerous white matter lesions, which is a typical biomarker of cerebral small vessel disease (SVD) on magnetic resonance images. The aim of the present study was to estimate the prevalence of CF in elderly Chinese patients with SVD. One hundred and thirty elderly patients with SVD were recruited for this cross-sectional observational study. Participants who met three to five of the Fried criteria of the physical frailty (PF) phenotype (shrinking, weakness, slowness, self-reported exhaustion, or low physical activity) were classified as having PF. Then, individuals with PF were defined as having CF if mild cognitive impairment was discovered by the Mini-Mental State Examination. Lastly, a series of cognitive function tests and the dual-task walking paradigm were examined. Based on the CF diagnostic criteria, the frequency of CF was 23.08% among elderly Chinese patients with SVD. Furthermore, CF-positive patients had a more significant SVD burden, based on magnetic resonance imaging findings. Logistic regression analysis, which was adjusted for age, sex, education, and comorbidities, showed that CF was negatively correlated with the dual-task walking speed in elderly people with SVD. Thus, SVD burden might be an indicator of CF phenotype. In elderly patients with SVD, CF was associated with dual task walking performance.

## Introduction

Owing to the global growth in the number of elderly people (1), frailty has become an increasingly important concept (2). Cognitive frailty (CF), defined as the presence of physical frailty (PF) and cognitive impairment in the absence of dementia, has recently been proposed as a distinct entity in geriatrics (3). Cognitive frailty has been associated with falls, functional decline, disability, dementia, and all-cause mortality in longitudinal studies (4–6). These factors result in an increasing burden on public health. However, research has confirmed that proper interventions can reverse or partially reverse CF (7–9). In clinically-based research conducted in different countries, the prevalence of CF among elderly people ranged from 10.7 to 40.0% (10). However, relevant data are lacking in mainland China.

For a long time, cognitive impairment and PF have been studied separately. In recent years, evidence has increasingly indicated that impairments in cognitive and physical dimensions are often concurrent (11). Approximately 20% of physically frail individuals may be cognitively impaired or vice versa (12, 13). These results imply that these two conditions share an overlaid pathology. As a result, several investigators have suggested new conceptual frameworks, such as CF and motoric cognitive risk syndrome, in their research, especially for Alzheimer's disease, Parkinson's disease, and many other neurodegenerative diseases (14).

Cerebral small vessel disease (SVD) is a syndrome that involves diseases of the small vessels in the brain, such as white matter hyperintensity lesions, cerebral microbleeds, and subcortical infarcts (15). Small vessel disease is quite common in the older population. The two most common complaints are gait abnormalities and cognitive deficits, specifically a decline in executive function and cognitive flexibility (16). Though executive dysfunction is considered as one of the most common cognitive symptoms, executive function was not measured in frailty instruments, as has been reviewed by Azzopardi et al. (1).

However, investigations of CF and the relevant phenotype in people with SVD have been rare. Therefore, the purpose of the present study was to investigate CF and the relevant phenotype in elderly Chinese patients with SVD, and to investigate the relationship between CF and impairments in executive function/cognitive flexibility.

## Materials and Methods

### Participants

We conducted a clinical cross-sectional observational study from February 1, 2018 to April 1, 2019 and recruited 130 elderly patients with SVD from the Department of Neurology at the Seventh Medical Center of PLA General Hospital (Beijing, China). Our study was approved by the Academic Ethics Committee of the Biological Sciences Division of the Seventh Medical Center of PLA General Hospital in Beijing, China.

**Figure 1:**
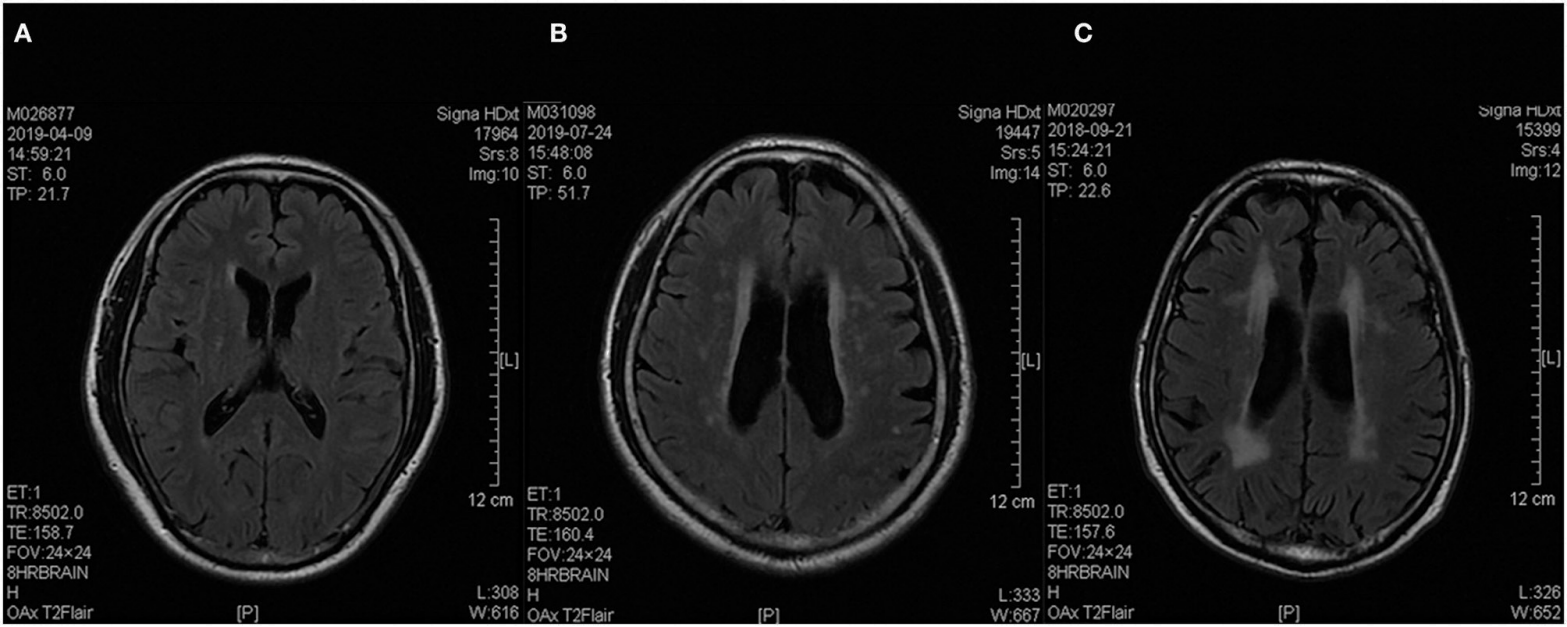
Sample brain T2 FLAIR MRI images of three patients. **(A–C)** Based on the Fazekas' score, Patients **(A–C)** were rated as 1, 2, and 3, respectively. They were grouped into the low burden SVD, moderate burden SVD, and high burden SVD groups, respectively. FLAIR, fluid-attenuated inversion recovery; MRI, magnetic resonance imaging, SVD, small vessel disease.

All patients were screened with a brain MRI on a 3 T scanner, and their SVD burden was evaluated and rated using Fazekas' scale (13, 17). The severity of white matter lesions was graded as “punctate lesions” (i.e., grade 1), “early confluent lesions” (i.e., grade 2), and “confluent lesions” (i.e., grade 3). Examples of MRI brain image findings of each group are presented in Figure 1.

The exclusion criteria were: patients with major stroke or cerebral bleeding episodes; other causes of leukoencephalopathy (e.g., immune, demyelination, genetic); major psychiatric diseases; use of psychotropic medications; multisystem diseases such as polyarteritis nodosa, nervous system vasculitis associated with connective tissue disorders, vasculitis secondary to infectious, etc.; arthritis; MRI contraindications; and dementia.

All participants had normal visual acuity and comprehensive capacity. The participants provided general information such as their name, sex, age, education level, and comorbidity number (hypertension, angina, myocardial infarction, hyperlipidemia, diabetes, stroke, migraine, arthritis, and falls history).

### Magnetic Resonance Imaging Measurements

A 3.0 T MRI scan (Discovery MR750; GE Healthcare, Waukesha, WI, USA) of the brain showed white matter lesions that were compatible with SVD grades 1, 2 or 3. The brain imaging protocol, based on a slice thickness of 5 mm and an interslice thickness of 1.5 mm, employed the following parameters: for T1 fluid-attenuated inversion recovery (FLAIR) images, the repetition time (TR) was 1,750 ms, echo time (TE) was 23 ms, TI was 780 ms, and field of view (FOV) was 24 cm; for T2-weighted images, TR was 7,498 ms, TE was 105 ms, and FOV was 24 cm.

### Definition of Physical Frailty and Cognitive Frailty

We defined PF based on the frailty phenotype proposed by Fried et al. (18) in the Cardiovascular Health Study. The components of the frailty phenotype include shrinking, weakness, slowness, self-reported exhaustion, and low physical activity. Shrinking was defined as unwanted weight loss of a minimum of 1 kg a week over two consecutive weeks or more, or a body mass index of <18.5 kg/m^2^ (19). Weakness was defined as low hand grip strength, as measured with JAMAR hand dynamometer (Jamar Plus +Digital 563213; Lafayette Instrument Company, Lafayette, IN, USA) of <18.0 kg for women and <26.0 kg for men (20). Single-task walking at usual speed was mentioned in the “Walking paradigm.” A slow walking speed was defined as a usual walking speed of <1.0 m/s, as measured by a wearable gait monitor (Intelligent Device for Energy Expenditure and Activity [IDEEA]) (20, 21). Exhaustion was defined as a negative response to the following question in the Geriatric Depression Scale (22): “Do you feel full of energy?” Low physical activity was defined as not engaging in physical exercise at least once weekly (23). Cognitive function was evaluated with the Mini-Mental State Examination. Participants with scores >27 points were categorized as “normal”; 19–26 points as having mild cognitive impairment; and <18 points as having dementia (20).

Participants who met three to five of the frailty phenotype criteria were classified as having PF (18). In this study, individuals with PF and mild cognitive impairment were considered as having CF (24). Individuals were otherwise considered as “non-CF patients.”

### Walking Paradigm for Single- and Dual-Task Walking

Elderly patients with SVD performed poorly under brain stress conditions. Therefore, an additional cognitive-motor dual task was established in addition to the single-task walking. Participants were asked to walk 25 strides in a long corridor at their usual (i.e., self-selected) speed under the single-task walking (STW) and dual-task walking (DTW) conditions. The start and possible end points for each trial were marked. The cognitive dual-task involved the individual performing serial 3 subtraction while walking with a starting number (chosen randomly) of 90, 95, 100, or 105, as described by Radovanović et al. (25). Previous research has shown that the walking performance under the double-task (DT) condition is associated with increased attention demands and with the risk of falls (26).

The participants were instructed to be as accurate as possible when performing the cognitive task. They were also reminded to continue walking for the full 25 strides. To avoid acceleration and deceleration effects, the participants started walking six steps before reaching the start line and completed their walk six steps beyond the 25th stride. The order of the single-task (ST) and DT gait conditions were presented randomly to avoid systematic bias. The dual-task cost (DTC) on ST speed and DT speed was calculated using the following equation (27):

$$\text{DTC}=-\frac{\text{dual task value }-\text{ single task value}}{\text{single task value}}*\;\text{100}$$

### Neuropsychological Assessment

All participants completed a series of neuropsychological assessments such as category verbal fluency test (VFT) (which reflects executive function) (28), clock drawing test (CDT) (which reflects visuospatial function) (29), and trail-making test–part B (which reflects cognitive flexibility) (30).

### Statistical Analysis

Differences between the groups' demographic data were analyzed by using one-way analysis of variance. The differences analyzed were on VFT, CDT, and TMT-B performance, as well as DTC. The Chi-squared test was used to compare the frequency of SVD burden between the CF and non-CF patients. Forward stepwise logistic regression was conducted to explore the association between various factors (i.e., the independent variables) and CF (i.e., the dependent variable). The analysis was adjusted for age, sex, education, and comorbidities. The data are expressed as the mean ± the standard deviation. A *P* < 0.05 was statistically significant. All statistical analyses were conducted using a statistical software package (SPSS, version 22.0; IBM Corp., Armonk, NY, USA).

## Results

Based on the Fried diagnostic criteria for physical frailty and the definition of CF, the frequency of CF among Chinese patients with SVD was 23.08% in this study. Table 1 shows the demographic characteristics of all patients. The CF-positive (CF+) group patients had a more severe SVD burden than did CF-negative (CF-) group patients (Fazekas score: 2.32 ± 0.68 vs. 1.56 ± 0.66; *P* = 0.000). Cognitive function differed significantly between the CF+ and CF- groups, as reflected by the scores on the VFT (13.98 ± 3.16 vs. 18.04 ± 3.58, *P* = 0.000), CDT (10.67 ± 1.32 vs. 11.97 ± 1.24, *P* = 0.000), trail-making test B (95.96 ± 18.20 vs. 72.46 ± 19.29, *P* = 0.000), and Mini-Mental State Examination (25.90 ± 1.09 vs. 28.11 ± 1.32, *P* = 0.000). However, the DTC was higher in the CF+ group than in the CF- group (19.73 ± 10.60 vs. 12.78 ± 8.46, *P* = 0.000).

**Table 1:** Clinical and demographic characteristics of the subjects with and without CF.

	**Overall** **(*N* = 130)**	**CF+** **(*N* = 30)**	**CF-** **(*N* = 100)**	***P*-value**
Men, %	80 (61.54%)	13 (56.67%)	67 (67.00%)	0.151
Age, years	66.78 (9.16)	69.87 (9.08)	63.25 (8.66)	0.000
Education, years Comorbidity number BMI, kg/m^2^	10.05 (3.61) 1.57 (0.99) 25.17 (3.26)	8.60 (3.64) 1.83 (1.12) 23.21 (3.70)	10.48 (3.50) 1.49 (0.94) 25.77 (2.89)	0.012 0.095 0.000
MMSE, score	27.60 (1.57)	25.90 (1.09)	28.11 (1.32)	0.000
VFT, words	17.10 (3.88)	13.98 (3.16)	18.04 (3.58)	0.000
CDT, score	11.67 (1.37)	10.67 (1.32)	11.97 (1.24)	0.000
TMT-B, seconds	77.88 (21.42)	95.96 (18.20)	72.46 (19.29)	0.0000
Fazekas score DTC	1.72 (0.72) 14.39 (9.43)	2.23 (0.68) 19.73 (10.60)	1.56 (0.66) 12.78 (8.46)	0.000^#^ 0.000

*Mean (Standard Deviation)*.

# *P < 0.05 CF+ relative to CF- CF+, with cognitive frailty; CF-, without cognitive frailty; BMI, Body Mass Index; MMSE, Mini-Mental State Evaluation; VFT, Verbal Fluency Test; CDT, Clock Drawing Test; TMT-B, Trail Making Test-B; DTC, Dual Task Cost*.

Furthermore, patients were grouped into one of three different levels of SVD burden, which were based on the MRI-based Fazekas score. As shown in Table 2, in Chinese patients with SVD, different frequencies of the PF phenotype between groups (high burden SVD vs. moderate burden SVD vs. low burden SVD) were detected for weakness (75.00 vs. 49.06 vs. 21.05%; *P* = 0.000), exhaustion (50.00 vs. 35.85 vs. 15.79%; *P* = 0.006), and slowness (90.00 vs. 71.70 vs. 33.33%; *P* = 0.000), but not for shrinkage (5.00 vs. 1.89 vs. 1.75%, *P* = 0.683) or low activity (50.00 vs. 47.17 vs. 36.84%, *P* = 0.437).

**Table 2:** Cognitive and motor function of the subjects with different levels of SVD.

	**Low burden SVD**	**Moderate burden SVD**	**High burden SVD**	***P*-value**
**Physical frailty component**				
Shrinkage, *N*(%) Weakness, *N*(%) Exhaustion, *N*(%) Low activity, *N*(%) Slowness, *N*(%) Fried Frailty, *N*(%)	1 (1.75) 12 (21.05) 9 (15.79) 21 (36.84) 19 (33.33) 9 (15.79)	1 (1.89) 26 (49.06) 19 (35.85) 25 (47.17) 38 (71.70) 19 (35.85)	1 (5.00) 15 (75.00) 10 (50.00) 10 (50.00) 18 (90.00) 12 (60.00)	0.683 0.000 0.006 0.437 0.000 0.001
**Cognitive function**				
MCI, *N*(%) VFT, words CDT, score TMT-B, seconds	12 (21.05) 19.21 (3.54) 12.33 (1.04) 65.35 (17.17)	32 (61.54) 16.21 (2.99) 11.45 (1.10) 82.83 (16.49)	17 (85.00) 13.45 (3.32) 10.35 (1.73) 100.50 (20.59)	0.000 0.000^*^^#^ 0.000^*^^#^ 0.000^*^^#^
**Motor function**				
DTW speed, m/s DTC	0.91 (0.19) 13.92 (9.39)	0.79 (0.22) 13.88 (9.49)	0.58 (0.20) 17.04 (9.38)	0.000^*^^#^ 0.395

* *P < 0.05 Moderate burden SVD relative to Low burden SVD individuals*.

# *P < 0.05 Severe-SVD relative to Moderate burden SVD individuals. SVD, Small Vessel Disease; MCI, Mild Cognitive Impairment; VFT, Verbal Fluency Test; CDT, Clock Drawing Test; TMT-B, Trail Making Test-B; DTW, Dual Task Walking; DTC, Dual Task Cost*.

However, we evaluated the cognitive and motor function of patients with different levels of SVD. In addition, the scores on the VFT (13.45 ± 3.32 vs. 16.21 ± 2.99 vs. 19.21 ± 3.43, *P* = 0.000), CDT (10.35 ± 1.73 vs. 11.45 ± 1.10 vs. 12.33 ± 1.04, *P* = 0.000), and trail-making test B (100.50 ± 20.59 vs. 82.83 ± 16.49 vs. 65.35 ± 17.17, *P* = 0.000), and the DTW speed (0.58 ± 0.20 m/s vs. 0.79 ± 0.22 m/s vs. 0.91 vs. 0.19 m/s, *P* = 0.000) were statistically different between groups. Details are presented in Table 2.

Furthermore, the association between CF and cognitive and motor function was explored by using logistic regression analysis, adjusted for age, sex, education and comorbidities. The CF was negatively correlated with DTW speed (*P* = 0.020, standardized β = −4.624) among Chinese patients with SVD. Details are presented in Table 3.

**Table 3:** Logistic regression for associated factors with CF.

	**Model 1**		**Model 2**	
	**Standardized** **β-value**	***P*****-value**	**Standardized** **β-value**	***P*****-value**
VFT	−0.077	0.604	0.036	0.825
CDT	0.310	0.345	0.275	0.444
TMT-B	0.037	0.209	0.035	0.306
DTW speed	−4.656	0.014^*^	−4.624	0.020^*^
DTC	0.042	0.166	0.046	0.140
Fazekas Score	0.457	0.309	0.894	0.087

*Model 1: Logistic regression for risk factors associated with CF. Model 2: Logistic regression for risk factors associated with CF. Adjusted for age, gender, education and comorbidities*.

* *P < 0.05. CF, Cognitive Frailty; VFT, Verbal Fluency Test; CDT, Clock Drawing Test; TMT-B, Trail Making Test-B; DTW, Dual Task Walking; DTC, Dual Task Cost*.

## Discussion

A recent study (31) reports that the standard prevalence of CF is 2.0% among community-dwelling old adults in mainland China. In the present study, we found that ~23.08% of elderly Chinese patients with SVD had CF. Furthermore, CF+ patients had a more severe SVD burden than did CF- patients. These findings were not in line with those of a study on elderly Indian individuals with SVD (32). In a study by Wang et al. (32), only cortical lacunar infarcts, rather than white matter lesions, were correlated with cognitive and motor dysfunctions. Empirically-based research has revealed that the SVD burden is associated with a wide range of clinical symptoms such as cognitive deficits, dysfunction in the upper and lower extremities, and low physical activity (33, 34). In the current study, which used Fried's diagnostic criteria for CF, our findings implied that SVD is closely correlated with CF.

Patients with SVD were further divided into three groups based on the MRI-based Fazekas score. The results demonstrated that elderly Chinese patients with SVD had a unique CF phenotype. As shown in Table 2, the PF phenotype differed between each group. The frequency of shrinkage was low and did not increase in accordance with the SVD burden, whereas the frequency of low activity was equally high between the three groups. The findings elucidated that elderly Chinese people with SVD rarely experienced body loss or lack of nutrition, but lacked exercise. For weakness, exhaustion, and slowness, the frequencies increased in line with the SVD burden. In addition, the rate of patients with mild cognitive impairment also increased with SVD burden. Consistent with previous results (23, 35), our findings indicated that white matter lesions may be associated with the pathologies of CF, especially with weakness, exhaustion, slowness, and the cognitive domain.

According to the Fried criteria of frailty, slow walking speed was one of the five phenotype components (slowness). So a low STW speed (at self-selected speed) is equivalent to slowness. Evidence has confirmed that gait is a complex activity that is as much a cognitive as a motor task (36), and DTW gait changes could be interpreted as resulting from impairment in executive function in prodromal Alzheimer's Disease patients (37). Logistic regression analysis revealed that the DTW speed was significantly and negatively correlated with CF. An explanation for this finding is that DTW is a simultaneous paradigm, which requires a high load of simultaneous cognitive and motor functions (38). DTW is a brain stress test that patients with SVD have difficulty in accomplishing (39). Cognitive tests or the STW speed cannot comprehensively reflect CF in elderly patients with SVD. Therefore, DTW speed could be a useful tool for indicating cognitive and motor dysfunction in SVD patients.

Several limitations of this study warrant consideration. First, the sample size was small. Second, a longitudinal study rather than a cross-sectional study may be more helpful for investigating CF and factors associated with CF progression in elderly subjects with SVD. Third, some aspects, such as memory and orientation ability, were not investigated in the present study because patients with SVD characteristically do not show deficits in these domains. In future research, we will overcome these limitations.

In conclusion, the prevalence of CF was quite high in elderly Chinese patients with SVD. Based on the MRI Fazekas score, CF+ patients had a more severe SVD burden. Cognitive frailty was negatively correlated with the DTW speed in elderly patients with SVD. The results elucidated that small vessel disease burden might be an indicator of the cognitive frailty phenotype. In elderly patients with small vessel disease, cognitive frailty was associated with dual-task walking performance.

## Data Availability Statement

All datasets generated for this study are included in the article/supplementary material.

## Ethics Statement

The studies involving human participants were reviewed and approved by Our study was approved by the Academic Ethic Committee of the Biological Sciences Division of the Seventh Medical Center of PLA General Hospital, Beijing, China. The patients/participants provided their written informed consent to participate in this study.

## Author Contributions

HZ and WW were responsible for data collection. YL was responsible for manuscript writing. JG was responsible for data analysis. YH was responsible for the study design. All authors contributed to the article and approved the submitted version.

## Conflict of Interest

The authors declare that the research was conducted in the absence of any commercial or financial relationships that could be construed as a potential conflict of interest.

## Abbreviations

CDT: clock drawing test
CF: cognitive frailty
DT: dual-task
DTC: dual-task cost
DTW: dual-task walking
MRI: magnetic resonance imaging
PF: physical frailty
ST: single-task
STW: single task walking
SVD: small vessel disease
VFT: verbal fluency test.
